# Food Substitution Modeling Approaches: A Methodological Study

**DOI:** 10.1016/j.cdnut.2026.109396

**Published:** 2026-06-13

**Authors:** Thomas Roosdorp, Michael Fridén, Daniel B Ibsen, Fredrik Rosqvist

**Affiliations:** 1 Department of Food Studies, Nutrition and Dietetics, Uppsala University, Uppsala, Sweden; 2 Department of Public Health and Caring Sciences, Clinical Nutrition and Metabolism, Uppsala University, Uppsala, Sweden; 3 Department of Clinical Medicine, Aarhus University, Aarhus, Denmark; 4 Steno Diabetes Center Aarhus, Aarhus University Hospital, Aarhus, Denmark; 5 Department of Public Health, Aarhus University, Aarhus, Denmark

**Keywords:** nutritional epidemiology, diet records, food substitution, substitution models, cohort studies, methodology

## Abstract

**Background:**

The interpretation of results from observational studies in nutrition depends on how food intake is modeled. When adjusting for total energy or total food intake, substitution effects are estimated. Simulation studies show that the choice of modeling approach for estimating substitution effects strongly influences the results, with some models producing estimates opposite to the true simulated effect. However, few studies have investigated these different approaches using empirical data.

**Objectives:**

We compared different substitution models using fermented and nonfermented milk as the exposure and all-cause mortality as the outcome in a Swedish cohort of n = 1063 elderly men.

**Methods:**

Two substitution modeling strategies were compared: a nonspecified approach (adjusting for total energy) and a specified approach (adjusting for total energy and other foods). The specified approach was divided into a leave-one-out model (excluding substituted food) and a comprehensive leave-one-out model (excluding substituted food while adjusting for remaining foods). We also examined the influence of unit of choice [grams, kilocalories (kcal), or mixed] across all models. All substitution models (*n* = 18) used mixed-unit (kcal and gram) and same-unit (either gram or kcal) approaches. Cox proportional hazard regression models were used to estimate hazard ratios (HR) and 95% confidence intervals (CI) for all-cause mortality from the Swedish National Death Registry.

**Results:**

Nonspecified substitutions for nonfermented milk varied by unit of choice, with the mixed-unit approach (HR: 1.18; 95% CI: 0.95, 1.48) differing from the same-unit (kcal) approach (HR: 1.00; 95% CI: 0.80, 1.25). Fermented milk showed no estimated differences. The specified substitution models provided similar estimates across the unit of choice.

**Conclusions:**

In nonspecified substitution modeling, estimates differed for nonfermented milk. Fermented milk showed similar differences in both the nonspecified and specified leave-one-out models. Although estimates were generally consistent across specified substitution models, each estimate represents a distinct research question, and making this explicit is advised for future studies.

## Introduction

Interpreting results from nutritional epidemiological studies is challenging, partly due to the compositional nature of dietary data (i.e., higher intake of one food is most often compensated by lower intakes of other foods to maintain energy balance) [[1], [2], [3]]. As such, it is common to assess dietary intake in relation to total energy intake (TEI). In addition, adjusting for TEI is widely recognized as necessary to account for confounding from common determinants of dietary intake, such as body size and metabolism of foods [1,3]. It is, however, not widely acknowledged that adjusting for TEI has implications for the interpretation of diet–disease associations from energy-contributing foods [4].

Observational studies of diet encounter several methodological challenges, including specifying the comparator food or foods because there is seldom a placebo in nutrition research. As such, the use of statistical substitution models has increased in popularity over the last decade to make the comparison explicit and thus estimates easier to interpret [4]. The, most often, unintended consequences of not clearly specifying the comparator food(s) might be a contributing factor to the somewhat conflicting findings observed in nutritional epidemiology [5,6]. In addition, different substitution modeling choices and even the unit of choice might play a significant role in the interpretation of the results [4,7,8]. For instance, a simulation-based study by Tomova et al. [8] in 2022 found that the regression coefficient for the replacement of fish with meat reversed its sign when using a mixed-unit approach (i.e., grams for the foods and kcal for the composite variable TEI) compared with a same-unit approach [i.e., the foods and the composite variable of TEI or total food intake (TFI) in the same unit]. Whether the conflicting results from Tomova et al.’s simulation-based study might extrapolate to real-world data and help explain somewhat conflicting findings of diet–disease relations in nutritional epidemiology is still uncertain.

## Nonfermented and fermented milk as illustrative examples

Nonfermented and fermented milk provide a relevant and timely example of the issue discussed above because their associations with all-cause mortality have been examined extensively in nutritional epidemiology, yet findings remain inconsistent [9,10]. Although some cohort studies have found that fermented milk products, such as yogurt, are associated with lower mortality risk, others have reported null or even positive associations [[10], [11], [12], [13], [14]]. Similarly, findings for nonfermented milk also vary, with some studies reporting no association and others suggesting increased risk at higher intakes [[10], [11], [12], [13], [14]]. These inconsistencies may partly reflect differences in exposure classification and model specification. For example, some studies compared high compared with low reported intakes, whereas others used food substitution models with different modeling approaches, each resulting in distinct interpretations and sometimes different results. This heterogeneity makes nonfermented and fermented milk a useful empirical setting for examining how substitution model choice and unit specification can influence effect estimates and interpretation in observational data.

Therefore, the aim of this methodological study was to investigate the impact of different food substitution modeling approaches, including the unit of choice, using cohort data. We explored the associations of intake of either nonfermented or fermented milk and the association of replacing nonfermented milk with fermented milk on all-cause mortality.

## Methods

### Empirical data

Data from the Uppsala Longitudinal Study of Adult Men (ULSAM) were used for this methodological study (see Supplemental Figure 1 for a flowchart). ULSAM is a cohort study of elderly men, initiated in 1970 with the aim of identifying metabolic risk factors for cardiovascular disease (CVD). A detailed description of ULSAM has been provided elsewhere [15,16]. For the current study, the population included *n* = 1221 men aged ∼70 y. The eligibility criteria included having complete food records, a TEI between 800 and 4200 kcal/ [17], and no history of cancer diagnosis [18]. Out of *n* = 1221, *n* = 1063 remained after exclusion (Supplemental Figure 1).

### Dietary assessment

Dietary intake was evaluated using a 7-d food record provided by the Swedish Food Agency (version 1990) [19]. Participants were given oral instructions by a dietitian on how to complete the food record. Foods not defined in the food record were reported as “other.” The food record was validated against an open-ended, weighed 7-d food record in a subset of the ULSAM cohort [20]. In addition, the 7-d food record has been validated against biomarkers using other versions of dietary food records [19]. Each food item was reported in grams and kilocalories (kcal).

We classified food items (*n* = 691) into the following 10 food categories: nonfermented milk, fermented milk, other dairy products, fruits and vegetables, cereals, red meat and processed meat, fish and shellfish, sweets and sugar-sweetened beverages, alcoholic beverages, and other foods (Supplemental Table 1).

Intake of each food item was imputed as 0 if there was no reported intake. Food group variables were calculated by summing all food items specified for that group in grams or kilocalories. TEI and TFI were calculated as the total sum of energy intake (in kcal) and food intake (in grams), respectively, from all reported food items. All dietary intake variables were further transformed and divided by 100 kcal or 200 g for energy intake and food intake, respectively, as these closely resemble a serving size. To compare high compared with low intake of either nonfermented or fermented milk, quartiles were calculated from continuous food group variables in grams and kcal. Records with implausible TEI (<800 or >4200 kcal/d) were excluded according to predefined criteria.

### Modeling approaches

The relative causal effect*,* that is, the effect on a health outcome if one food was substituted for another [1,3], is often a target estimand in nutritional studies. Researchers may achieve this by using statistical food substitution models, keeping TEI fixed [4,21]. There are 2 main types of substitution models commonly used in the field of nutrition: a nonspecified substitution approach, which aims to estimate the relative causal effect of substituting a specific food item with a concomitant lower intake of the background diet; and a specified substitution approach, which aims to estimate the relative causal effect of substituting a food item with a concomitant lower intake of a specified comparator food (or foods) [4]. One widely used specified substitution method is the leave-one-out model (a variation of the standard model for the nonspecified substitution) that involves adjusting for TEI and a composite food group that includes the exposure food of interest, as well as other food items (e.g., total milk as a composite food group including both nonfermented and fermented milk), while excluding the intended food to be replaced (e.g., nonfermented milk), hence leave-one-out [3,4]. An extension of the leave-one-out approach involves adjusting for TEI or TFI and all other food items except for the food to be replaced. This particular version is hereafter referred to as the comprehensive leave-one-out approach [4,8]. In other words, in the leave-one-out model, the comparator is a composite of the exposure and selected related foods, whereas in the comprehensive leave-one-out model, the comparator includes all remaining food items except the exposure being replaced.

The choice among these approaches should be guided by the research question, whether the goal is to better understand broader substitution patterns in observational data or to mimic a targeted food replacement intervention. Recognizing these distinctions clarifies why different models may yield different results and helps identify which model is most appropriate for a given research purpose.

### Unit of choice

Tomova et al. [8] showed in their simulation study that the unit of choice impacts the resulting estimates. However, choosing the appropriate unit (e.g., kilojoules and kcal for energy, grams for weight, or volumes and servings for other units) for the chosen food substitution model is often not rationalized by researchers, and the implications of not doing so are not well understood [8,22]. In this study, we used both a mixed-unit approach and a same-unit approach, the former more commonly used in observational studies of diet.

### Model specifications

The different versions of the food substitution models were selected because of their common use in nutritional epidemiological studies. All model specifications were prespecified before conducting the analyses (*n* = 18 different models; Table 1). The model specifications included:

1) Nonfermented or fermented milk as a continuous exposure.
2) High compared with low intake of either nonfermented milk or fermented milk (i.e., categorical).
3) Leave-one-out approach (i.e., substituting nonfermented milk with fermented milk and adjusting for TEI and total milk as a composite variable).
4) The comprehensive leave-one-out approach (i.e., substituting nonfermented milk with fermented milk and adjusting for TEI and all other foods).

**TABLE 1:** Model specifications and interpretations of various target estimands for the different substitution models.

Model^1^	Specification^2^	Estimand	Interpretation
Nonspecified substitution			
Exposure as continuous			
Mixed-unit^5^	$\log\left(h\left(t;x\right)\right)=\log\left(h_{0}\left(t\right)\right)+\beta_{1}{\text{Milk}/\text{Fermented}\;\text{Milk}}_{gram}+\beta_{2}\text{TEI}+\beta_{3}\text{covariates}$	The relative causal effect of a 200 g higher intake of milk/fermented milk, holding total energy intake (kcal) constant.	$\exp\left(\beta_{1}\right)$ represents the estimated difference in hazard rates between persons with a 200 g higher intake of nonfermented milk/fermented milk, with substitution with the background diet, within the same amount of total energy.
Gram^5^	$\log\left(h\left(t;x\right)\right)=\log\left(h_{0}\left(t\right)\right)+\beta_{1}{\text{Milk}/\text{Fermented}\;\text{Milk}}_{gram}+\beta_{2}\text{TFI}+\beta_{3}\text{covariates}$	The relative causal effect of a 200 g higher intake of milk/fermented milk, holding total food intake (grams) constant.	$\exp\left(\beta_{1}\right)$ represents the estimated difference in hazard rates between persons with a 200 g higher intake of nonfermented milk/fermented milk, with substitution with the background diet. The substitution is hence an equal mass substitution and does not guarantee an isocaloric substitution.
Kcal^5^	$\log\left(h\left(t;x\right)\right)=\log\left(h_{0}\left(t\right)\right)+\beta_{1}{\text{Milk}/\text{Fermented}\;\text{Milk}}_{kcal}+\beta_{2}\text{TEI}+\beta_{3}\text{covariates}$	The relative causal effect of a 100 kcal higher intake milk/fermented milk, holding total energy intake (kcal) constant.	$\exp\left(\beta_{1}\right)$ represents the estimated difference in hazard rates between persons with a 200 g higher intake of nonfermented milk/fermented milk, with substitution with the background diet, within the same amount of total energy. The substitution is isocaloric.
High vs. low intake of either milk or fermented milk^3^			
Mixed-unit^4^^and^^5^	$\log\left(h\left(t;x\right)\right)=\log\left(h_{0}\left(t\right)\right)+\beta_{1}{\text{Milk}/\text{Fermented}\;\text{Milk}}_{Q2}^{}+\beta_{2}{\text{Milk}/\text{Fermented}\;\text{Milk}}_{Q3}^{}+\beta_{3}{\text{Milk}/\text{Fermented}\;\text{Milk}}_{Q4}^{}+\beta_{4}\text{TEI}+\beta_{5}\text{covariates}$	The relative causal effect of high vs. low milk/fermented milk intake (in grams), holding total energy intake (kcal) constant.	$\exp\left(\beta_{3}\right)$ represents the estimated difference in hazard rates between persons of high vs. low intake (Q4 vs. Q1), with substitution with the background diet, within the same amount of total energy.
Same-unit (gram)^4^^and^^5^	$\log\left(h\left(t;x\right)\right)=\log\left(h_{0}\left(t\right)\right)+\beta_{1}{\text{Milk}/\text{Fermented}\;\text{Milk}}_{Q2}^{}+\beta_{2}{\text{Milk}/\text{Fermented}\;\text{Milk}}_{Q3}^{}+\beta_{3}{\text{Milk}/\text{Fermented}\;\text{Milk}}_{Q4}^{}+\beta_{4}\text{TFI}+\beta_{5}\text{covariates}$	The relative causal effect of high vs. low milk/fermented milk intake (grams), holding total food intake (grams) constant.	$\exp\left(\beta_{3}\right)$ represents the estimated difference in hazard rates between persons of high vs. low intake (Q4 vs. Q1), with substitution with the background diet. The substitution is hence an equal mass substitution and does not guarantee an isocaloric substitution.
Same-unit (kcal)^4^^and^^5^	$\log\left(h\left(t;x\right)\right)=\log\left(h_{0}\left(t\right)\right)+\beta_{1}{\text{Milk}/\text{Fermented}\;\text{Milk}}_{Q2}^{}+\beta_{2}{\text{Milk}/\text{Fermented}\;\text{Milk}}_{Q3}^{}+\beta_{3}{\text{Milk}/\text{Fermented}\;\text{Milk}}_{Q4}^{}+\beta_{4}\text{TEI}+\beta_{5}\text{covariates}$	The relative causal effect of high vs. low milk/fermented milk intake (kcal), holding total energy intake (kcal) constant.	$\exp\left(\beta_{3}\right)$ represents the estimated difference in hazard rates between persons of high vs. low intake (Q4 vs. Q1), with substitution with the background diet, within the same amount of total energy. The substitution is isocaloric.
Specified substitution			
Leave-one-out approach			
Mixed-unit^5^^and^^6^	$\log\left(h\left(t;x\right)\right)=\log\left(h_{0}\left(t\right)\right)+\beta_{1}{\text{Milk}}_{\text{fermented}}+\beta_{2}{\text{Milk}}_{\text{total}}^{}+\beta_{3}\text{TEI}+\beta_{4}\text{covariates}$	The relative causal effect of fermented milk instead of nonfermented milk (grams), holding total energy intake (kcal) constant.	$\exp\left(\beta_{1}\right)$ represents the estimated difference in hazard rates between persons with a 200 g higher intake of fermented milk and a simultaneously equal lower intake of nonfermented milk within the same level of TEI and total milk intake. Adjusting for TEI however will introduce a residual energy substitution when the exposure is modeled in grams and the 2 foods differ in energy density.
Same-unit (gram)^5^^and^^6^	$\log\left(h\left(t;x\right)\right)=\log\left(h_{0}\left(t\right)\right)+\beta_{1}{\text{Milk}}_{\text{fermented}}+\beta_{2}{\text{Milk}}_{\text{total}}^{}+\beta_{3}\text{TFI}+\beta_{4}\text{covariates}$	The relative causal effect of fermented milk instead of nonfermented milk (grams), holding total food intake (grams) constant.	$\exp\left(\beta_{1}\right)$ represents the estimated difference in hazard rates between persons with a 200 g higher intake of fermented milk and a simultaneously equal lower intake of nonfermented milk within the same level of TFI and total milk intake. The equal mass substitution does not guarantee isocaloric substitutions if the 2 foods differ in energy density.
Same-unit (kcal)^5^^and^^6^	$\log\left(h\left(t;x\right)\right)=\log\left(h_{0}\left(t\right)\right)+\beta_{1}{\text{Milk}}_{\text{fermented}}+\beta_{2}{\text{Milk}}_{\text{total}}^{}+\beta_{3}\text{TEI}+\beta_{4}\text{covariates}$	The relative causal effect of fermented milk instead of nonfermented milk (kcal), holding total energy intake (kcal) constant.	$\exp\left(\beta_{1}\right)$ represents the estimated difference in hazard rates between persons with a 100 kcal higher intake of fermented milk and a simultaneously equal lower intake of nonfermented milk within the same level of TEI and total milk intake. The substitution is isocaloric.
Comprehensive			
leave-one-out approach			
Mixed-unit^5^^and^^7^	$\log\left(h\left(t;x\right)\right)=\log\left(h_{0}\left(t\right)\right)+\beta_{1}{\text{Milk}}_{\text{fermented}}+\beta_{2}\text{TEI}+\beta_{3}\text{covariates}+\beta_{4}\text{other}\;\text{foods}$	The relative causal effect of fermented milk instead of nonfermented milk (grams), for the same amount of total energy (kcal), holding all other foods constant.	$\exp\left(\beta_{1}\right)$ represents the estimated difference in hazard rates between persons with a 200 g higher intake of fermented milk and simultaneously equal lower intake of nonfermented milk within the same level of intake from other foods included in the model and TEI. Introduces restrictions of the underlying dietary patterns.
Same-unit (gram)^5^^and^^7^	$\log\left(h\left(t;x\right)\right)=\log\left(h_{0}\left(t\right)\right)+\beta_{1}{\text{Milk}}_{\text{fermented}}+\beta_{2}\text{TFI}+\beta_{3}\text{covariates}+\beta_{4}\text{other}\;\text{foods}$	The relative causal effect of fermented milk instead of nonfermented milk (in grams), for the same amount of total food (grams), holding all other foods constant.	$\exp\left(\beta_{1}\right)$ represents the estimated difference in hazard rates between persons with a 200 g higher intake of fermented milk and a simultaneously equal lower intake of nonfermented milk within the same level of intake from other foods and TFI. The substitution is equal mass.
Same-unit (kcal)^5^^and^^7^	$\log\left(h\left(t;x\right)\right)=\log\left(h_{0}\left(t\right)\right)+\beta_{1}{\text{Milk}}_{\text{fermented}}+\beta_{2}\text{TEI}+\beta_{3}\text{covariates}+\beta_{4}\text{other}\;\text{foods}$	The relative causal effect of fermented milk instead of nonfermented milk (kcal), for the same amount of total energy (kcal), holding all other foods constant.	$\exp\left(\beta_{1}\right)$ represents the estimated difference in hazard rates between persons with a 100 kcal higher intake of fermented milk and a simultaneously equal lower intake of nonfermented milk within the same level of intake from other energy-providing foods and TEI. The substitution is isocaloric.

Abbreviations: kcal, kilocalories; Q, quartile; TEI, total energy intake; TFI, total food intake.

1 Substitutions are expressed in 100 kcal/d or in 200 g/d (makes estimates comparable as 200 g of dairy ≈ 100 kcal).

2 Cox proportional hazards regression model, i.e., log(hazard at time *t*).

3 High vs. low intake is compared in quartiles as well as in 100 kcal/d units.

4 Being quartiles of either nonfermented milk or fermented milk, expressed in grams or kcal.

5 Covariates being age, physical activity, education, BMI, sleep, stress, current smoking, family history of cardiovascular disease, family history of diabetes, and feeling of loneliness.

6 Total milk being a composite of nonfermented milk and fermented milk, expressed in grams or kcal.

7 Other foods being fruits and vegetables, cereals, red meat and processed red meat, fish and shellfish, sweets and sugar-sweetened beverages, alcoholic beverages, and other foods.

Each modeling approach also considered the type of units used in the model specification, where 3 additional approaches for the unit of choice were used: a mixed-unit approach, in which TEI is measured in kcal and food intake in grams; a same-unit (gram) approach, in which TFI (instead of TEI) and food intake are measured in grams; and a same-unit (kcal) approach, in which TEI and food intake are measured in kcal.

### Statistical analysis

Hazard ratios (HR) and their corresponding 95% confidence intervals (CIs) were estimated using a multivariable Cox proportional hazard regression model, with time since baseline (in years) serving as the underlying timescale. The time at risk of all-cause mortality was calculated from the date of the baseline visit (1991–1995) to the date of death or administrative end of follow-up (31 December, 2011), whichever occurred first.

Confounders were chosen a priori based on subject matter knowledge, a previous adjustment set for the ULSAM cohort [23], previous literature on nutritional epidemiological adjustment sets [7,24], and the use of a directed acyclic graph (DAG) created using DAGitty [24]. The following confounders were selected: age, physical activity, education, BMI, sleep, stress, current smoking, family history of CVD, family history of diabetes, and feelings of loneliness (as a proxy for single-person household, which may in turn influence dietary intake) (Supplemental Figure 2).

Our primary analysis used multiple imputation to address missing data, assuming that the data were missing at random or missing completely at random [25]. Any covariates that included “do not know” as answers were coded as missing. The imputation was performed using Multiple Imputations by Chained Equations with 20 imputations and 10 iterations [26]. All analyses were conducted using R Statistical Software (version 4.3.2) [27] and RStudio (version 2023.12.1) [28].

## Results

A total of *n* = 1063 participants were included. Of these, *n* = 723 died during the 20-y follow-up period. Baseline characteristics of the cohort are presented in Supplemental Table 2. Dietary intake measured at baseline is presented in Supplemental Table 3 for the whole cohort as well as stratified by high and low intakes of nonfermented and fermented milk. The difference in the mean intake of nonfermented milk between those with high (Q4) and low (Q1) intake was ∼462 g or 219 kcal, and for fermented milk it was 236 g or 125 kcal. Individuals with the highest intake (Q4) of nonfermented milk had the highest total dairy intake with 595.9 g/d, and those with the lowest intake (Q1) of nonfermented milk had the lowest total dairy intake with 180.3 g/d. The total cohort contributed 15,343.9 y of follow-up, with a median follow-up time of 12.5 y. See Supplemental Table 4 for a description of the outcome in terms of counts and crude rates for the whole cohort and by quartile of the exposures.

### Nonspecified substitution modeling approaches

#### Exposure as continuous

The estimates from the nonspecified substitution approach, using the exposure on a continuous scale for both nonfermented and fermented milk, were similar across the different approaches in unit of choice (Table 2). For nonfermented milk, using the mixed-unit approach, the HR was 1.05 (95% CI: 0.96, 1.14), for the same-unit approach (gram): 1.02 (95% CI: 0.94, 1.11), and for the same-unit approach (kcal): 1.04 (95% CI: 0.95, 1.14). For fermented milk, using the mixed-unit approach, the HR was 0.86 (95% CI: 0.73, 1.00), for the same-unit approach (gram): 0.85 (95% CI: 0.73, 1.00), and for the same-unit (kcal) it was 0.87 (95% CI: 0.75, 1.01).

**TABLE 2:** Results of 18 model specifications using multivariable Cox regression with multiple imputed data (*n* = 1063).

Model	Mixed unit^1^		Same unit (g)		Same unit (kcal)	
	HR	95% CI	HR	95% CI	HR	95% CI
Nonspecified substitution						
Exposure as continuous^2^						
Nonfermented milk^3^	1.05	0.96, 1.14	1.02	0.94, 1.11	1.04	0.95, 1.14
Fermented milk^3^	0.86	0.73, 1.00	0.85	0.73, 1.00	0.87	0.75, 1.01
High vs. low intake of nonfermented milk^2^						
Q1	Ref	Ref	Ref	Ref	Ref	Ref
Q2	0.95	0.77, 1.18	0.94	0.76, 1.16	0.89	0.72, 1.10
Q3	1.01	0.81, 1.26	0.98	0.79, 1.22	1.07	0.86, 1.33
Q4	1.18	0.95, 1.48	1.12	0.90, 1.39	1.00	0.80, 1.25
High vs. low intake of fermented milk^2^						
Q1	Ref	Ref	Ref	Ref	Ref	Ref
Q2	0.88	0.72, 1.09	0.89	0.72, 1.10	0.97	0.79, 1.20
Q3	0.94	0.77, 1.15	0.94	0.76, 1.15	0.89	0.72, 1.09
Q4	0.82	0.66, 1.01	0.81	0.66, 1.01	0.80	0.64, 0.99
Specified substitution						
Leave-one-out approach^4^						
Fermented milk^3^	0.84	0.71, 0.99	0.85	0.72, 1.00	0.85	0.73, 0.98
Comprehensive leave-one-out approach^5^						
Fermented milk^3^	0.83	0.70, 0.97	0.80	0.68, 0.95	0.87	0.74, 1.02

Modeled as exposure on a continuous scale using the standard multivariate method; high vs. low intake; leave-one-out or comprehensive leave-one-out approach for the substitution of nonfermented milk with fermented milk. Covariates being age, physical activity, education, BMI, sleep, stress, current smoking, family history of cardiovascular disease, family history of diabetes, and feeling of loneliness.

Abbreviations: CI, confidence interval; HR, hazard ratio; Q, quartile; TEI, total energy intake; TFI, total food intake.

1 Food variables are measured in grams, whereas TEI is measured in kcal.

2 Adjusted for TEI/TFI and covariates.

3 Measured either as per 100 kcal or 200 g.

4 Adjusted for TEI/TFI, total milk, and covariates.

5 Adjusted for TEI/TFI, other foods, and covariates.

#### Exposure as categorical

Estimates for the nonspecified substitution comparing high compared with low intake of nonfermented milk were different across the different approaches in unit of choice, albeit imprecise and with overlapping CI (Table 2). Using the mixed-unit approach, the HR was 1.18 (95% CI: 0.95, 1.48), for the same-unit approach (gram): 1.12 (95% CI: 0.90, 1.39), and for the same-unit approach (kcal): 1.00 (95% CI: 0.80, 1.25). For the nonspecified substitution of high compared with low intake of fermented milk, similar estimates were found between the different approaches of units. Using the mixed-unit approach, the HR was 0.82 (95% CI: 0.66, 1.01), for the same-unit approach (gram): 0.81 (95% CI: 0.66, 1.01), and for the same-unit approach (kcal): 0.80 (95% CI: 0.64, 0.99).

### Specified substitution modeling approaches

Using the leave-one-out approach for the substitution of nonfermented milk with fermented milk, estimates were comparable between the different unit-of-choice approaches with an HR of 0.84 (95% CI: 0.71, 0.99) for the mixed-unit approach, 0.85 (95% CI: 0.72, 1.00) for the same-unit approach (gram), and 0.85 (95% CI: 0.73, 0.98) for the same-unit approach (kcal) (Table 2). For the comprehensive leave-one-out approach, the HR for the mixed-unit approach was 0.83 (95% CI: 0.70, 0.97), for the same-unit approach (gram): 0.80 (95% CI: 0.68, 0.95), and for the same-unit approach (kcal): 0.87 (95% CI: 0.74, 1.02).

## Discussion

This methodological study explored how different food substitution modeling approaches commonly used in nutritional epidemiology influence estimates of the comparison between nonfermented milk and fermented milk and all-cause mortality. The specified substitutions produced consistent results across the different units, suggesting that the unit choice has little impact on the results obtained from the cohort data when clearly specifying the comparison food. In contrast, however, results from the nonspecified substitution models for nonfermented milk showed different estimates across the different units.

### Nonspecified substitution modeling approaches

A notable finding was that the nonspecified substitution of high compared with low intake of nonfermented milk produced different estimates when using a mixed-unit compared with a same-unit (kcal) approach. The same pattern across the different models was not observed for fermented milk or for nonfermented milk when the exposure was continuous. These discrepancies may partly reflect differences in background diet between quartile groups, as suggested by the descriptive food data. The difference in total volume between the highest compared with lowest category of nonfermented milk represented roughly 2 servings, whereas the corresponding contrast for fermented milk represented only ∼1 serving. This difference highlights the difference in dietary patterns regarding food items and the specific eating contexts of foods that may not even be comparable between different countries or cultural populations [21]. For example, in a Swedish setting, nonfermented milk is frequently added to hot beverages or substituted with other drinks, whereas fermented milk is more often consumed with cereals at breakfast or as a snack. Ultimately, estimates from nonspecified food substitution models reflect the substitution of an isolated food with a mix of all other foods, as determined by the background diet of the study population. As a result, these estimates are more sensitive to changes in measurement units and may not generalize well to other populations with different eating patterns, because variations in the background diet can meaningfully alter the substitution effect.

Similar patterns have been reported in other methodological work. MacDonald et al. [22] compared mixed-unit and same-unit substitution models across dietary quartiles using processed meat as the exposure and observed higher HRs for the highest intake group under mixed-unit specification (HR: 1.14; 95% CI: 1.08, 1.21) compared with the same-unit approach (HR: 1.08; 95% CI: 1.02, 1.14). Collectively, our and previous findings suggest that mixed-unit models can alter the magnitude of the estimated effects, but the extent to which this may be applied to other cohort studies needs to be investigated further.

### Specified substitution modeling approaches

In contrast to the heterogeneity observed for the nonspecified substitutions of nonfermented milk, the specified leave-one-out models produced consistent estimates across the different approaches. Conceptually, specified substitution models statistically mimic research questions posed in strictly controlled dietary interventions, where the exchange between 2 foods occurs under stable total energy conditions [3]. The leave-one-out approach is the more common approach and estimates the relative effect of replacing one food with another while maintaining TEI, whereas the comprehensive approach does so while additionally controlling for all other foods in the diet [4]. Thus, the 2 models answer closely related but not identical questions, which helps explain why their estimates may slightly differ. Although these models target different research questions, they each support a clearer formulation of causal questions by making assumptions explicit and estimands better defined.

Supporting our findings, MacDonald et al. [22] reported only modest differences between leave-one-out models and the comprehensive leave-one-out models when substituting fatty fish with processed meat (although these were called energy partition models in the article but are mathematically the same as the leave-one-out models). Similarly, Ibsen et al. [29] showed that estimates for several specified food replacements across different substitution models and units were somewhat stable, although for some signs actually reversed when comparing the mixed-unit approach to the same-unit approach (kcal). Speculatively, larger differences between models may be expected when comparing foods with more divergent nutritional or energy profiles. Taken together, these findings suggest that when the substitution is specified, the choice of modeling framework exerts less influence on the estimated effect. Importantly, however, the interpretations of these estimates will differ (i.e., different research questions are being answered; see Table 1).

A limitation of the comprehensive leave-one-out approach is that its interpretation depends on how completely the dietary composition is represented in the model. In our analysis, food items were classified into broad food groups, but some foods were grouped into broader categories or residual “other foods” rather than modeled separately. Consequently, the estimated substitution effect should be interpreted conditionally on the food structure included in the model, rather than as a fully exhaustive replacement of all foods in the diet.

### Strengths and limitations

A strength of this study is the application of multiple substitution models to a common dietary exposure and outcome, allowing direct evaluation of how model specification, unit of choice, and exposure classifications influence the resulting estimates. The use of prespecified modeling strategies and explicit interpretations of these strategies enhances transparency and interpretability, helping to clarify sources of divergence. However, several limitations warrant consideration. First, dietary intake was assessed at a single time point, limiting inference about long-term intake. Second, measurement error in both dietary and confounding variables, as well as unobserved confounding, may have biased our results. Third, these results are based on a single cohort study, thus warranting replication in other studies.

### Implications for practitioners

This study highlights key principles for designing and interpreting food substitution analyses in observational nutrition research. First, researchers should begin with a clearly defined causal question and specify the target estimand, such as whether the aim is to estimate an isocaloric substitution effect or the total effect of increased intake [4,8]. Second, identifying such effects requires explicit assumptions about exchangeability, measurement error, and consistency, which should be evaluated in context [30]. Third, model specification must be transparent, including the exposure, comparator, and modeling approach (e.g., energy partition or leave-one-out). These choices shape the estimand and its interpretability [8]. For example, high compared with low intake comparisons may appear straightforward but often imply ambiguous substitutions. Fourth, the unit of exposure should also align with the research question and practical relevance [8]; although gram-based models support compositional interpretations, serving-based units may better inform dietary guidance. In many studies, however, servings/d are simply derived from grams/d using a fixed serving size and therefore may be broadly comparable with gram-based expressions unless serving definitions vary substantially across contexts. Finally, if a well-defined food substitution cannot be fully specified, due to a mismatch in units or unmeasured covariates, researchers should explain the rationale for the chosen approach and clarify what the model is estimating. Together, these principles provide a framework for generating interpretable and relevant estimates in food substitution research using observational data.

In conclusion, our findings suggest that the choice of substitution modeling approach may not always have a strong influence on the resulting estimates themselves (with nonfermented milk for the nonspecified quartile comparisons as the exception), but the choice of modeling framework will influence the interpretation of such estimates. In addition, the compositional nature of dietary data complicates the interpretation of the classic nonspecified mixed-unit approach. Researchers should carefully guide the modeling framework, including the unit of choice, by the underlying research question, and not the other way around. Such alignment will not only enhance comparability across studies but also, hopefully, lead to more meaningful questions being asked.

## Author contributions

The authors’ responsibilities were as follows – MF, FR: designed research; TR: analyzed data and wrote the manuscript; FR: primarily responsible for final content; and all authors: read and approved the final manuscript.

## Data availability

Data described in the manuscript, code book, and analytic code will be made available upon request.

## Declaration of Generative AI and AI-assisted technologies in the writing process

The authors declare that no generative AI or AI-assisted technologies were used in the writing of this manuscript.

## Funding

The authors reported no funding received for this study.

## Conflict of interest

The authors report no conflicts of interest.
