# Supplementary material for: Food Substitution Modeling Approaches: A Methodological Study
Source: Curr Dev Nutr. 2026 Jun 13;10(7):109396. doi: 10.1016/j.cdnut.2026.109396 (PMC13380801; doi:10.1016/j.cdnut.2026.109396)
Supplement: multimedia component 1 [file mmc1.docx]

Food substitution modelling approaches: a methodological study

Thomas Roosdorp^1*^, Michael Fridén^2,3,4^, Daniel B. Ibsen^4,5^, Fredrik Rosqvist^1,2^

1 Uppsala University, Department of Food studies, Nutrition and Dietetics

2 Uppsala University, Department of Public Health and Caring Sciences, Clinical Nutrition and Metabolism

3 Department of Clinical Medicine, Aarhus University

4 Steno Diabetes Center Aarhus, Aarhus University Hospital, Aarhus, Denmark

5 Department of Public Health, Aarhus University, 8000 Aarhus, Denmark

***** Correspondence: Thomas Roosdorp, thomas.roosdorp@ikv.uu.se, 0046768140429

| **Supplementary Table 1.** Categorization of food items into food groups. | |
| --- | --- |
| **Food group** | **Food items** |
| Non-fermented milk* | Milk <0.1% fat, milk 0.5% fat, milk 1.5% fat, milk 3% fat, milk 4.2% fat. |
| Fermented milk* | Sour milk 0.5% fat, sour milk 1.5% fat, sour milk 3% fat, yoghurt 3% fat. |
| Dairy other | Butter, brunost (eng. ‘brown cheese’), cottage cheese, quark, granular cheese, processed cheese, camembert, brie, parmesan, sour cream, cream, crème fraiche, etc. |
| Fruits and vegetables | Citrus fruits, stone fruits, berries, kiwi, root vegetables (except for potato), cruciferous vegetables, nightshade vegetables, pulses, legumes, cabbages, etc. |
| Cereals | Flour, wheat, rice, oats, crackers, pasta, breads, crispbreads, porridges, etc. |
| Red meat and processed red meat | Game meat, pork, beef, lamb, offal, bacon, sausages, etc. |
| Fish and shellfish | Salmon, sardines, tuna, eel, mackerel, herring, cod, crayfish, shrimp, lobster, crab, etc. |
| Sweets and sugar-sweetened beverages | Flavored milk, flavored fermented milk, canned fruit, breakfast cereals, cookies, cake, pastries, kissel, puddings, mousse, pies, marmalades, jams, chocolates, candy, sugar, honey, etc. |
| Alcoholic beverages | Beer, cider, red wine, white wine, spirits and liqueurs, etc. |
| Other foods | Margarine, vegetable oils, mayonnaise, potato, white meat (chicken, turkey, etc.), eggs, caviars, blood foods, nuts, snacks, water, coffee, tea, condiments, complete dishes, soups, sauces, etc. |
| * Only unflavored/unsweetened variants are included. | |

**Supplementary Table 2.** Baseline characteristics of participants in the *Uppsala Longitudinal Study of Adult Men* cohort.

| **Characteristics** | **Cohort** | **Non-fermented  milk Q1** | **Non-fermented milk Q4** | **Fermented  milk Q1** | **Fermented**  **milk Q4** |
| --- | --- | --- | --- | --- | --- |
| Participants (N) | 1063 | 257 | 271 | 271 | 258 |
|  | **Mean (SD)** | **Mean (SD)** | **Mean (SD)** | **Mean (SD)** | **Mean (SD)** |
| Age (years) | 71.0 (0.6) | 71.0 (0.6) | 71.0 (0.7) | 71.2 (0.5) | 71.0 (0.6) |
| BMI (kg/m^2^) | 25.3 (3.1) | 25.1 (2.9) | 25.4 (3.4) | 25.6 (3.1) | 24.8 (2.9) |
|  | **N (%)** | **N (%)** | **N (%)** | **N (%)** | **N (%)** |
| Education level |  |  |  |  |  |
| ≤ 7 years (elementary school) | 600 (56.4) | 111 (43.2) | 178 (65.7) | 167 (61.6) | 111 (43.0) |
| 8-13 years (secondary school) | 307 (28.9) | 90 (35.0) | 60 (22.1) | 75 (27.7) | 91 (35.3) |
| > 13 years (university studies) | 156 (14.7) | 55 (21.4) | 32 (11.8) | 29 (10.7) | 53 (20.5) |
| Family history of diabetes | 152 (14.3) | 25 (9.8) | 43 (15.9) | 36 (13.3) | 41 (15.9) |
| Family history of CVD | 624 (58.7) | 149 (58.2) | 167 (61.6) | 162 (59.8) | 152 (58.9) |
| Physical active | 627 (59.0) | 141 (55.1) | 161 (59.4) | 151 (55.7) | 154 (59.7) |
| Current smoker | 210 (19.8) | 45 (17.6) | 67 (24.7) | 74 (27.3) | 42 (16.3) |
| Trouble sleeping | 113 (10.6) | 21 (8.2) | 36 (13.3) | 28 (10.3) | 31 (12.0) |
| Feeling stressed | 144 (13.5) | 35 (13.7) | 37 (13.7) | 29 (10.7) | 40 (15.5) |
| Feeling of loneliness |  |  |  |  |  |
| very seldom/never | 612 (57.6) | 162 (63.0) | 157 (57.9) | 164 (60.6) | 140 (54.3) |
| seldom | 366 (34.4) | 77 (30.0) | 88 (32.8) | 84 (31.0) | 92 (35.7) |
| often | 39 (3.7) | 7 (2.7) | 10 (3.7) | 11 (4.1) | 14 (5.4) |
| nearly always | 8 (0.8) | 2 (0.8) | 0.0 (0.0) | 2 (0.7) | 1 (0.4) |
| Q = Quartile, SD = Standard deviation, BMI = Body mass index, CVD = Cardiovascular disease | | | | | |

**Supplementary Table 3.** Dietary intake at baseline for the *Uppsala Longitudinal Study of Adult Men* cohort.

|  | **Cohort** | **Non-fermented  milk Q1** | **Non-fermented milk Q4** | **Fermented  milk Q1** | **Fermented milk Q4** |
| --- | --- | --- | --- | --- | --- |
|  | Median (IQR) | Median (IQR) | Median (IQR) | Median (IQR) | Median (IQR) |
| Total Food Intake (g/d) | 2224.3 (742.8) | 2051.3 (545.4) | 2404.1 (826.3) | 2167.4 (759) | 2379.5 (779.5) |
| Total Energy Intake (kcal/d) | 1699.2 (584.5) | 1574.0 (510.4) | 1962.0 (646.3) | 1700 (581.0) | 1832.1 (575.6) |
| Total dairy (g/d) | 335.8 (258.6) | 180.3 (190.2) | 595.9 (200.0) | 306.2 (248.8) | 464.8 (239.0) |
| Total dairy (kcal/d) | 271.5 (171.5) | 189.2 (133.7) | 378.9 (166.2) | 250.9 (161.1) | 334.9 (166.1) |
| Non-fermented milk (g/d) | 200.0 (257.1) | 27.7 (57.1) | 490.1 (142.9) | 267.8 (257.1) | 187.8 (228.6) |
| Non-fermented milk (kcal/d) | 94.0 (118.4) | 10.9 (25.7) | 201.1 (83.4) | 111.0 (116.0) | 67.1 (110.0) |
| Fermented milk (g/d) | 57.1 (157.1) | 114.6 (200.0) | 66.4 (114.3) | 0.0 (0.0) | 237.0 (78.6) |
| Fermented milk (kcal/d) | 29.9 (80.3) | 49.2 (94.8) | 16.2 (61.7) | 0.0 (0.0) | 115.0 (51.2) |
| Milk other (g/d) | 34.3 (29.66) | 38.0 (30.9) | 39.4 (32.1) | 38.4 (30.7) | 40.0 (28.6) |
| Milk other (kcal/d) | 120.3 (103.8) | 111.4 (113.7) | 126.1 (112.7) | 122.6 (115.4) | 122.6 (102.9) |
| Fruits and vegetables (g/d) | 112.1 (123.2) | 134.3 (122.0) | 131.0 (133.9) | 119.5 (127.7) | 137.3 (114.1) |
| Fruits and vegetables (kcal/d) | 55.6 (75.2) | 56.0 (75.0) | 48.0 (73.5) | 45.2 (72.9) | 60.5 (78.0) |
| Cereals (g/d) | 186.7 (142.0) | 152.7 (82.0) | 268.3 (161.3) | 223.9 (165.6) | 188.8 (99.6) |
| Cereals (kcal/d) | 408.1 (212.1) | 356.9 (210.1) | 460.7 (229.7) | 422.1 (217.5) | 406.0 (201.3) |
| Red meat (g/d) | 34.3 (28.6) | 37.6 (24.2) | 39.8 (28.6) | 40.4 (27.4) | 35.5 (26.6) |
| Red meat (kcal/d) | 81.8 (70.1) | 80.6 (61.6) | 81.3 (80.7) | 85.3 (69.7) | 75.5 (64.8) |
| Fish and shellfish (g/d) | 0.0 (4.3) | 4.0 (5.9) | 3.4 (1.1) | 4.0 (5.1) | 4.2 (5.9) |
| Fish and shellfish (kcal/d) | 0.0 (7.8) | 0.0 (14.5) | 0.0 (4.6) | 0.0 (10.4) | 0.0 (11.0) |
| Sweets and SSB (g/d) | 105 (149.4) | 124.5 (135.5) | 147. 0 (153.5) | 130.6 (127.1) | 171.4 (176.5) |
| Sweets and SSB (kcal/d) | 181.6 (210.2) | 150.8 (184.7) | 193.5 (214.9) | 163.9 (177.4) | 212.0 (237.4) |
| Alcoholic beverages (g/d) | 133.7 (214.3) | 228.6 (257.1) | 116.0 (171.4) | 170.8 (230.5) | 177.4 (222.6) |
| Alcoholic beverages (kcal/d) | 38.0 (68.2) | 57.4 (91.0) | 24.6 (55.2) | 35.2 (69.6) | 39.7 (64.9) |
| Other foods (g/d) | 1169.5 (525.4) | 1294.9 (580.0) | 1285.3 (522.7) | 1256.0 (507.2) | 1301.6 (498.0) |
| Other foods (kcal/d) | 568.2 (223.2) | 540.3 (216.4) | 617.2 (233.5) | 562.4 (213.0) | 576.6 (192.5) |
| Q = Quartile, IQR = Interquartile range, SSB = Sugar sweetened beverages | | | | | |

| **Supplementary Table 4.** Number of events and crude rates of all-cause mortality for total (n = 1063), stratified by quartiles of non-fermented milk and fermented milk intake. | | | |
| --- | --- | --- | --- |
| **Variable** | **Person-years** | **All-cause mortality  (n)** | **Rate/1000  Person-Years** |
| Total | 15343.9 | 723 | 47.1 |
| Non-fermented milk |  |  |  |
| Q1 | 3788.6 | 178 | 47.0 |
| Q2 | 4054.6 | 180 | 44.4 |
| Q3 | 3765.9 | 174 | 46.2 |
| Q4 | 3734.8 | 191 | 51.1 |
| Fermented milk |  |  |  |
| Q1 | 3714.3 | 200 | 53.9 |
| Q2 | 3862.2 | 173 | 44.8 |
| Q3 | 3916.9 | 189 | 48.3 |
| Q4 | 3850.4 | 161 | 41.8 |
| Q = Quartile | | | |


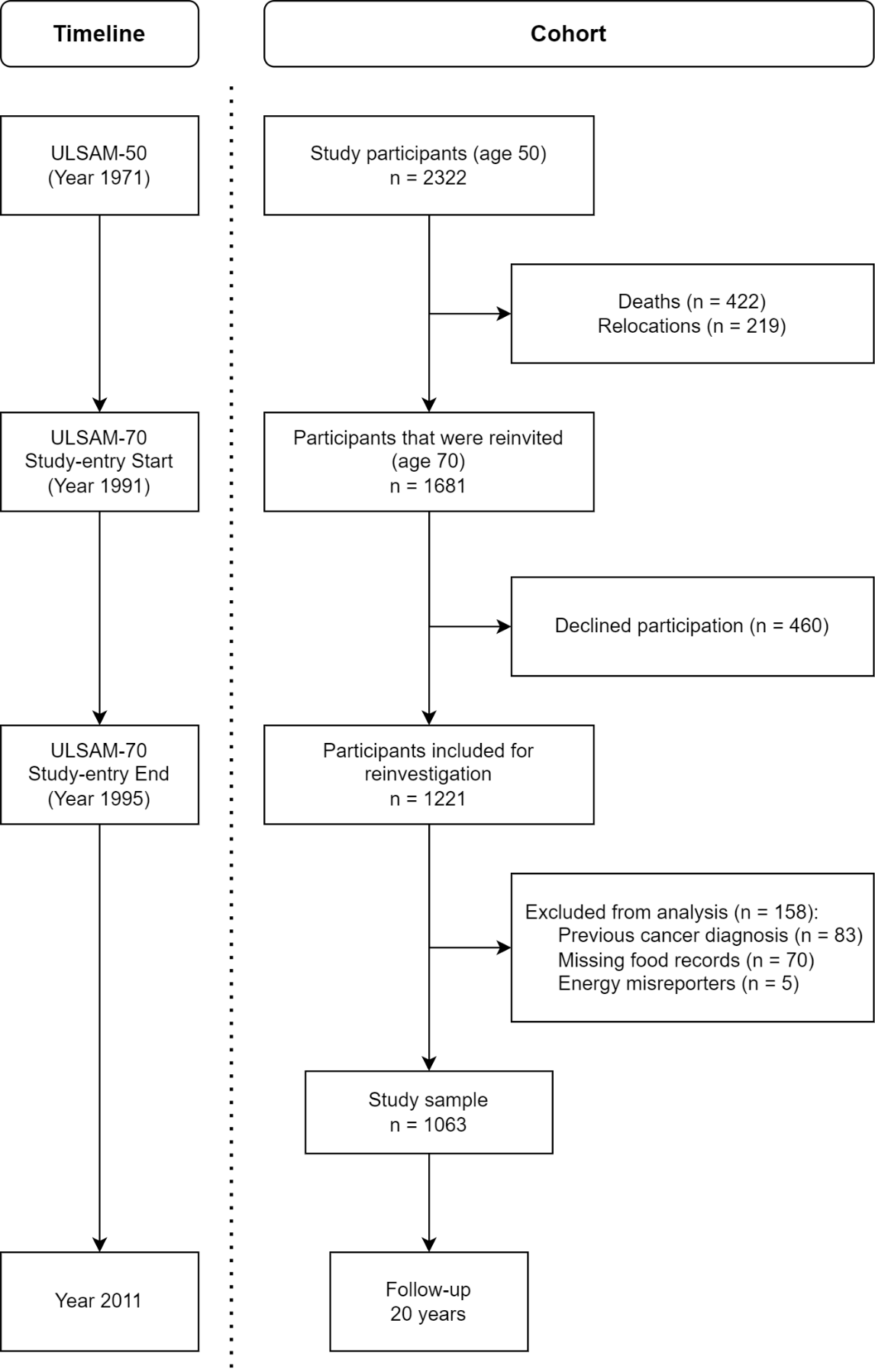


**Supplementary Figure 1**. Flowchart describing the timeline and cohort for the ULSAM study, starting from the ULSAM-50 cohort, and ending with end of follow-up for the ULSAM-70 cohort. ULSAM being the Uppsala Longitudinal Study of Adult Men.


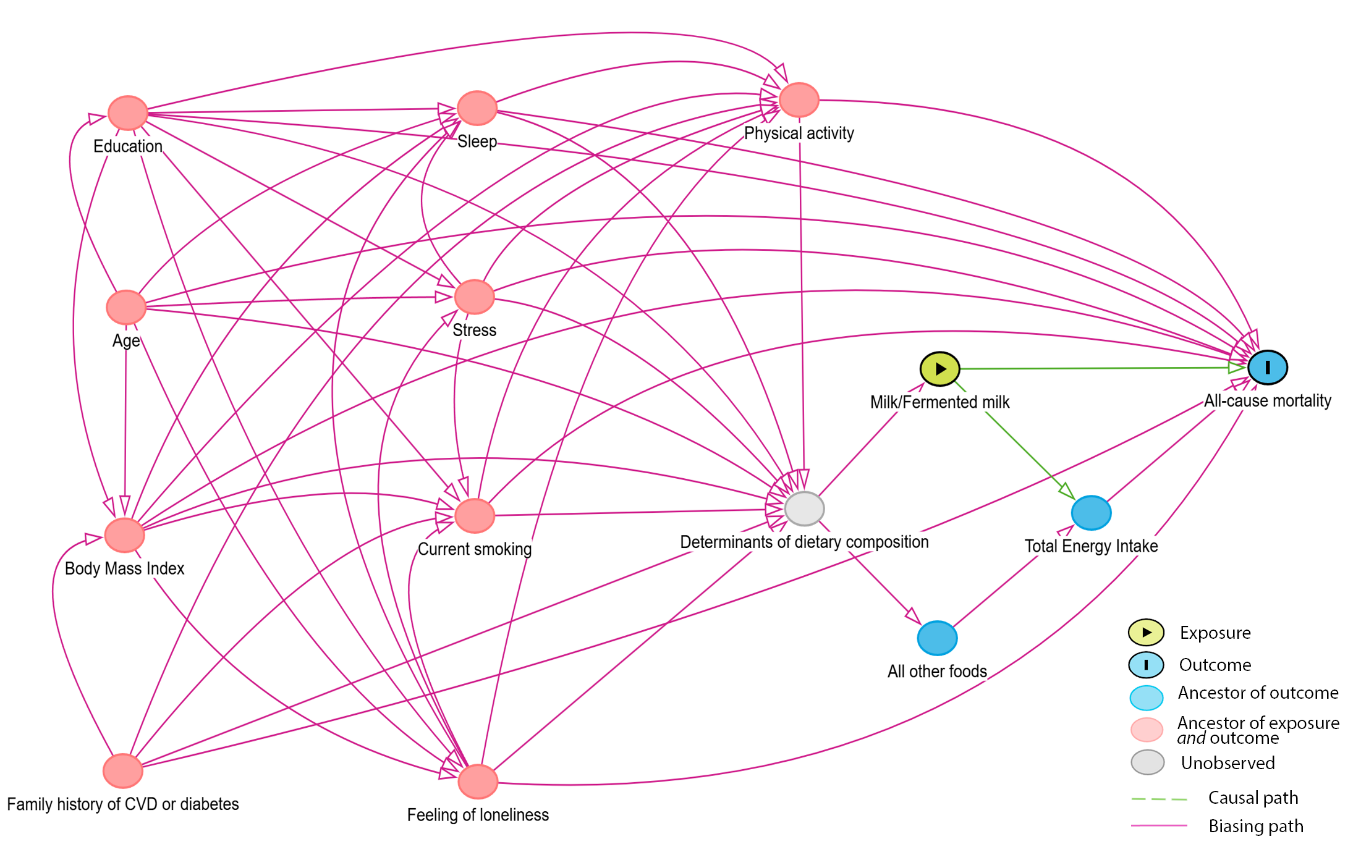


**Supplementary Figure 2.** A general overview of a directed acyclic graph (DAG) showing our causal assumptions between milk/fermented milk as exposure and all-cause mortality as the outcome. CVD, cardiovascular disease.
